# Prior osteosynthesis—unlike osteotomy—raises revision risk after total knee arthroplasty, predominantly via periprosthetic infection

**DOI:** 10.1002/ksa.70153

**Published:** 2025-10-28

**Authors:** Nele Wagener, Yinan Wu, Alexander Grimberg, Christian Hipfl, Sebastian Hardt

**Affiliations:** ^1^ Center for Musculoskeletal Surgery, Charité‐University Medicine Berlin Berlin Germany; ^2^ EPRD Deutsche Endoprothesenregister gGmbH Berlin Germany

**Keywords:** national registry study, osteosynthesis, osteotomy, periprosthetic joint infection, revision risk, total knee arthroplasty

## Abstract

**Purpose:**

Previous knee surgery is discussed as a risk factor for total knee arthroplasty (TKA), yet most reports do not distinguish between osteotomy and osteosynthesis. The German Operationen‐ und Prozedurenschlüssel (OPS)‐based classification was used to compare the impact of these procedures on implant survival, mortality and complications after primary TKA.

**Methods:**

Data from the German Arthroplasty Registry (2012–2024) were used. Patients with prior osteosynthesis (*n* = 10,078) and prior osteotomy (*n* = 96,782) were each 1:1 Mahalanobis‐matched to controls without prior knee surgery (matching on age, sex, Elixhauser score, body mass index [BMI], fixation, arthroplasty type and bearing mobility). Primary endpoints were all‐cause revision and mortality; secondary endpoints were periprosthetic joint infection (PJI), periprosthetic fracture and aseptic loosening. Kaplan–Meier with 95% log–log confidence intervals (CI) and log‐rank tests were used; Cox proportional hazards models estimated hazard ratios (HRs).

**Results:**

All‐cause revision differed for osteosynthesis versus controls (log‐rank *p* < 0.001) but not for osteotomy (*p* = 0.972). In Cox models, prior osteosynthesis was associated with a higher revision hazard (HR 1.81, 95% CI 1.52–2.15; *p* < 0.001). For osteotomy, no increased revision hazard was observed across age (<65/≥65 years) or comorbidity (Elixhauser < 3/≥3) strata. Mortality was higher after osteosynthesis (HR 1.48, 95% CI 1.34–1.64; *p* < 0.001), while osteotomy showed no overall increase; PJI occurred more often after osteosynthesis (HR 1.62, 95% CI 1.21–2.19). Periprosthetic fracture (HR 8.54, 95% CI 3.02–24.1) and aseptic loosening (HR 2.38, 95% CI 1.24–4.59) were likewise increased after osteosynthesis.

**Conclusions:**

Prior osteosynthesis increases revision risk after TKA—predominantly via infection—whereas prior osteotomy does not. These findings support extended perioperative infection workup, staged hardware removal when indicated and careful implant selection in patients with prior osteosynthesis; outcomes after osteotomy are comparable to primary osteoarthritis.

**Level of Evidence:**

Level IV, retrospective observational study.

**Abbreviations**
BMI: body mass index
CI: confidence interval
EPRD: Endoprothesenregister Deutschland (German Arthroplasty Registry)
HR: hazard ratio
HTO: high tibial osteotomy
ICD‐10‐GM: International Statistical Classification of Diseases, 10th Revision—German Modification
OA: osteoarthritis
OPS: Operationen‐ und Prozedurenschlüssel (German Operation and Procedure Classification)
PJI: periprosthetic joint infection
PPF: periprosthetic fracture
PROMs: patient‐reported outcome measures
SD: standard deviation
SMD: standardised mean difference
TKA: total knee arthroplasty
UKA: unicompartmental knee arthroplasty
VVC: varus–valgus constrained (knee prosthesis)

## INTRODUCTION

Total knee arthroplasty (TKA) is an established option for advanced knee osteoarthritis (OA) when conservative measures have been exhausted [7]. Secondary OA frequently arises after conditions or procedures that alter joint structure—often in the context of osteotomies or osteosyntheses [7, 15]. While osteotomies are typically used in younger patients to correct varus/valgus malalignment and restore the mechanical axis, thereby delaying arthroplasty [6, 9], osteosyntheses serve to stabilise periarticular fractures of the distal femur or proximal tibia [2, 12].

Patients regularly present for TKA after previous osteosynthesis or osteotomy. Nevertheless, decisions regarding infection workup, timing of hardware removal (staged vs. simultaneous) and choice of implant constraint still largely rely on indirect evidence: prior registry and cohort studies mostly examined either post‐traumatic/osteosynthesis‐associated cases [4, 15] or TKA after osteotomy [3, 5, 8, 13, 14], rather than comparing both interventions head‐to‐head within a unified methodological framework.

Operationen‐ und Prozedurenschlüssel (German Operation and Procedure Classification) (OPS) 2025 coding in the Endoprothesenregister Deutschland (German Arthroplasty Registry) (EPRD) was used to distinguish osteosynthesis from osteotomy and to compare both exposures in parallel, each with 1:1 Mahalanobis‐matched control groups.

For the first time, all relevant endpoints (revision, mortality, periprosthetic joint infection [PJI], fracture and aseptic loosening) are analysed within a consistent methodological setting, including stratified analyses by implant constraint and by age/comorbidity strata.

### Primary hypothesis

Prior osteosynthesis (vs. no prior knee surgery) increases the hazard of all‐cause revision and all‐cause mortality within ≤8 years after primary TKA, whereas prior osteotomy (vs. no prior surgery) does not; consistency: the osteosynthesis effect applies to both constrained and unconstrained implants, while the osteotomy null holds across age (<65/≥65 years) and comorbidity (Elixhauser < 3/≥3) strata.

### Secondary hypotheses

Prior osteosynthesis is associated with higher hazards of PJI, periprosthetic fracture (PPF) and aseptic loosening; prior osteotomy shows no increases in these endpoints.

## METHODS

### Data source

For this retrospective cohort study, all primary TKAs recorded in the EPRD between 1 November 2012 and 30 September 2024 were included.

The EPRD links statutory insurer billing, an implant product database and hospital case reports; it covers approximately 70% of hip and knee arthroplasties in Germany and—via insurer linkage—provides near‐complete follow‐up for revisions and mortality. Ethics approval was obtained from Kiel University (D 473/11).

### Study population and endpoints

Of the 534,698 primary TKAs (2012–2024), two 1:1 Mahalanobis‐matched cohorts were created according to prior procedure: osteosynthesis (*n* = 10,078) and osteotomy (*n* = 96,782). Each was matched to controls without prior knee surgery on age, sex, Elixhauser score, BMI, fixation, arthroplasty type and bearing mobility.

Exposure definitions used OPS (version 2025) and outcome definitions used ICD‐10‐GM; full code lists are provided in the Supporting Information S1 and S2.

Primary endpoints were all‐cause revision (exchange or removal of ≥1 component) and all‐cause mortality; secondary endpoints were PJI, PPF and aseptic loosening.

### Classification of prior operations

OPS codes for osteosynthesis and osteotomy were grouped by anatomical location (distal femur, proximal tibia and patella). Detailed line‐by‐line code lists are provided in the Supporting Information S3 and S4.

### Statistical analysis

Two separate 1:1 Mahalanobis‐matched comparisons were analysed (osteosynthesis vs. control; osteotomy vs. control). Covariate balance was assessed using standardised mean difference (SMD < 0.1).

Time‐to‐event outcomes were evaluated using Kaplan–Meier estimates with 95% log–log confidence intervals and log‐rank tests (follow‐up up to 8 years); Cox proportional hazards regression reported hazard ratios (HRs) with 95% confidence intervals.

Prespecified analyses included constrained versus unconstrained implants and, for the osteotomy comparison, sensitivity analyses plus stratified Cox models by age (<65 vs. ≥65 years) and comorbidity (Elixhauser < 3 vs. ≥3). Socio‐economic variables, rehabilitation setting and follow‐up/education content are not captured by the registry and could not be adjusted for. Analyses were conducted in R (v4.4.2); two‐sided *p* < 0.05 was considered statistically significant.

### Power and sample size

The study was adequately powered: for osteosynthesis, the matched sample (*n* = 5039 per arm; 10,078 total) exceeded requirements derived from observed 8‐year revision‐free survival and event‐based checks; for osteotomy (*n* = 48,391 per arm; 96,782 total), minimal detectable HRs at 80% power were approximately 1.14–1.08 across 2%–5% incidence scenarios. Full calculations are provided in the Supporting Information S5.

## RESULTS

### Patient flow and baseline characteristics

**Figure 1:**
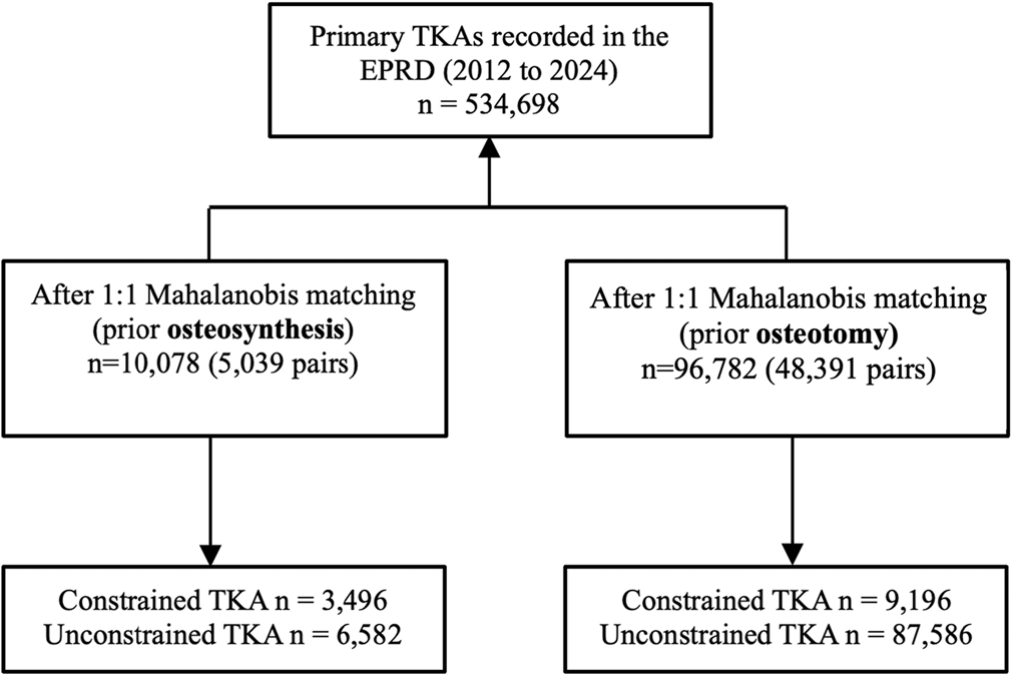
Study flow and cohort construction (EPRD, 2012–2024). EPRD, Endoprothesenregister Deutschland (German Arthroplasty Registry); TKA, total knee arthroplasty.

After 1:1 Mahalanobis‐matching, the osteosynthesis comparison comprised 5039 versus 5039 patients and the osteotomy comparison 48,391 versus 48,391; baseline characteristics of the matched groups were closely balanced across the matching variables (Table 1; Figure 1).

**Table 1:** Descriptive characteristics of the 1:1 Mahalanobis‐matched cohorts.

Characteristics	No prior osteosynthesis *n* = 5039/prior osteosynthesis *n* = 5039	Difference (SMD)	No prior osteotomy *n* = 48,391/prior osteotomy *n* = 48,391	Difference (SMD)
Sex		0		0
Female	3623 (71.9%)/3624 (71.9%)		32,299 (66.7%)/32,299 (66.7%)	
Male	1416 (28.1%)/1415 (28.1%)		16,092 (33.3%)/16,092 (33.3%)	
Age	70.9 (11.1) (28.0–98.0)/70.9 (11.3) (19.0–97.0)	0	69.6 (9.6) (24.0‐97.0)/69.5 (9.7) (21.0–98.0)	0
BMI		0		0
Underweight (<18.5)	56 (1.1%)/56 (1.1%)		134 (0.3%)/134 (0.3%)	
Normal (18.5–24.99)	1063 (21.1%)/1064 (21.1%)		6798 (14.0%)/6800 (14.1%)	
Pre‐obese (25.0–29.99)	1481 (29.4%)/1479 (29.4%)		13,877 (28.7%)/13,875 (28.7%)	
Obese 1 (30.0–34.99)	857 (17.0%)/857 (17.0%)		10,928 (22.6%)/10,928 (22.6%)	
Obese 2 (35.0–39.99)	355 (7.0%)/355 (7.0%)		5219 (10.8%)/5221 (10.8%)	
Obese 3 (≥40)	210 (4.2%)/210 (4.2%)		2751 (5.7%)/2751 (5.7%)	
Missing	1017 (20.2%)/1018 (20.2%)		8684 (17.9%)/8682 (17.9%)	
Elixhauser score	2.8 (5.8) (−8.0 to 42.0)/2.8 (5.9) (−8.0 to 42.0)	−0.01	0.9 (4.3) (−11.0 to 37.0)/0.9 (4.3) (−11.0‐40.0)	0
Fixation		0.12		0
Cemented	4814 (95.5%)/4693 (93.1%)		47,179 (97.5%)/47,170 (97.5%)	
Cementless	61 (1.2%)/61 (1.2%)		357 (0.7%)/357 (0.7%)	
Hybrid	162 (3.2%)/278 (5.5%)		833 (1.7%)/842 (1.7%)	
Reverse‐hybrid	2 (0.0%)/7 (0.1%)		22 (0.0%)/22 (0.0%)	
Type of arthroplasty		0		0
Constrained	1748 (34.7%)/1748 (34.7%)		4598 (9.5%)/4598 (9.5%)	
Unconstrained	3291 (65.3%)/3291 (65.3%)		43,793 (90.5%)/43,793 (90.5%)	
Bearing mobility		0		0
Fixed	4569 (90.7%)/4569 (90.7%)		44,047 (91.0%)/44,047 (91.0%)	
Mobile	470 (9.3%)/470 (9.3%)		4344 (9.0%)/4344 (9.0%)	

*Note*: Values are mean (standard deviation [SD]) or *n* (%). Age is shown as mean (SD), range. Difference = SMD. Baseline characteristics (1:1 Mahalanobis‐matched cohorts). Groups: with versus without prior osteosynthesis (*n* = 5039 each) and with versus without prior osteotomy (*n* = 48,391 each). Values: mean (SD) or *n* (%). Balance summarised by SMD.

Abbreviations: BMI, body mass index; SMD, standardised mean difference.

### Primary endpoints

**Figure 2:**
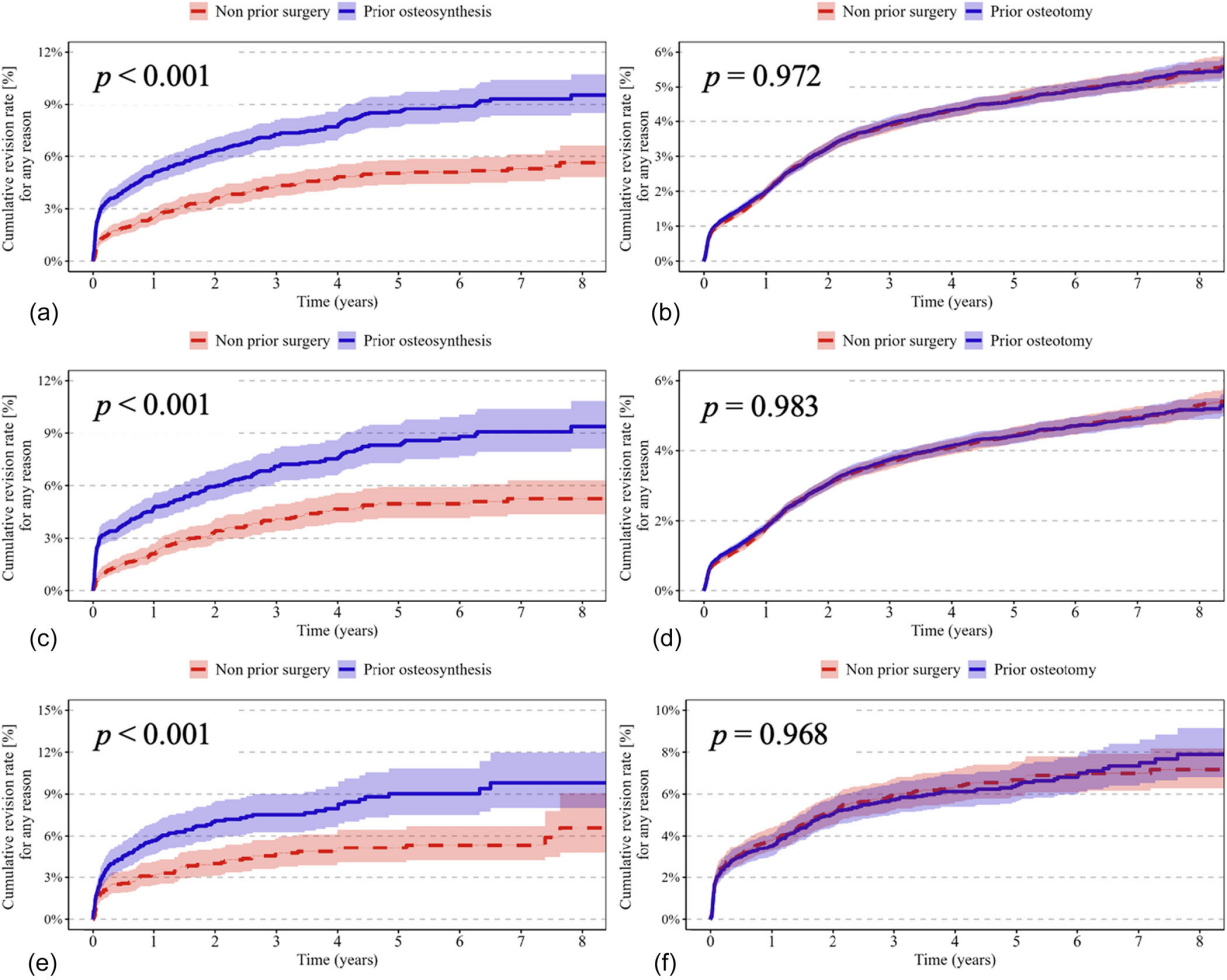
All‐cause revision after total knee arthroplasty (TKA) (Kaplan–Meier; follow‐up ≤ 8 years). Comparison: prior osteosynthesis or osteotomy versus matched controls. Panels (letters): a and b = all TKA (a, osteosynthesis; b, osteotomy); c and d = unconstrained (c, osteosynthesis; d, osteotomy); e and f = constrained (e, osteosynthesis; f, osteotomy). Shaded areas: 95% CI; *p*‐values: log‐rank. CI, confidence interval.

All‐cause revision: Prior osteosynthesis showed a higher revision risk with consistent effects in constrained and unconstrained TKAs (Figure 2; Table 2). Prior osteotomy showed no difference versus controls across prespecified age and comorbidity strata (Table 3).

**Table 2:** Cox proportional hazards estimates for outcomes after total knee arthroplasty (TKA) in patients with prior osteosynthesis versus matched controls.

Characteristic	Revision for any reasons (HR, 95% CI, *p*)	Mortality (HR, 95% CI, *p*)	Infection (HR, 95% CI, *p*)	PPF (HR, 95% CI, *p*)	Loosening (HR, 95% CI, *p*)
Osteosynthesis	1.81 (1.52–2.15), *p* < 0.001	1.48 (1.34–1.64), *p* < 0.001	1.62 (1.21–2.19), *p* < 0.001	8.54 (3.02–24.1), *p* < 0.001	2.38 (1.24–4.59), *p* = 0.009

*Note*: Outcomes after prior osteosynthesis versus matched controls (Cox proportional hazards models). Outcomes: all‐cause revision, mortality, infection, PPF and aseptic loosening. Values: HR (95% CI, *p*). Follow‐up ≤ 8 years; HR > 1 indicates a higher hazard in the osteosynthesis group.

Abbreviations: CI, confidence interval; HR, hazard ratio; PPF, periprosthetic fracture.

**Table 3:** Age‐ and comorbidity‐stratified Cox proportional hazards estimates for outcomes after total knee arthroplasty (TKA) in patients with prior osteotomy versus matched controls.

Group	Characteristic	<65 (HR, 95% CI, *p*)	≥65 (HR, 95% CI, *p*)	<3 (HR, 95% CI, *p*)	≥3 (HR, 95% CI, *p*)
Revision for any reasons	Osteotomy	1.05 (0.95–1.17), *p* = 0.362	0.97 (0.89–1.05), *p* = 0.435	1.00 (0.93–1.08), *p* = 0.955	0.99 (0.88–1.12), *p* = 0.900
Mortality	Osteotomy	0.97 (0.85–1.10), *p* = 0.619	0.94 (0.90–0.99), *p* = 0.015	0.96 (0.91–1.02), *p* = 0.223	0.90 (0.85–0.96), *p* = 0.002
Infection	Osteotomy	1.18 (0.95–1.46), *p* = 0.135	1.00 (0.87–1.16), *p* = 0.966	1.05 (0.91–1.22), *p* = 0.504	1.06 (0.86–1.30), *p* = 0.567
PPF	Osteotomy	1.12 (0.38–3.34), *p* = 0.836	0.81 (0.50–1.31), *p* = 0.389	0.79 (0.45–1.36), *p* = 0.389	0.99 (0.47–2.08), *p* = 0.979
Loosening	Osteotomy	0.94 (0.69–1.28), *p* = 0.697	0.81 (0.60–1.09), *p* = 0.157	0.84 (0.66–1.08), *p* = 0.172	0.97 (0.62–1.51), *p* = 0.888

*Note*: Stratified Cox models for prior osteotomy versus matched controls. Strata: age (<65/≥65) and Elixhauser index (<3/≥3). Outcomes: all‐cause revision, mortality, infection, PPF and aseptic loosening. Values: HR (95% CI, *p*); follow‐up ≤ 8 years.

Abbreviations: CI, confidence interval; HR, hazard ratio; PPF, periprosthetic fracture.

**Figure 3:**
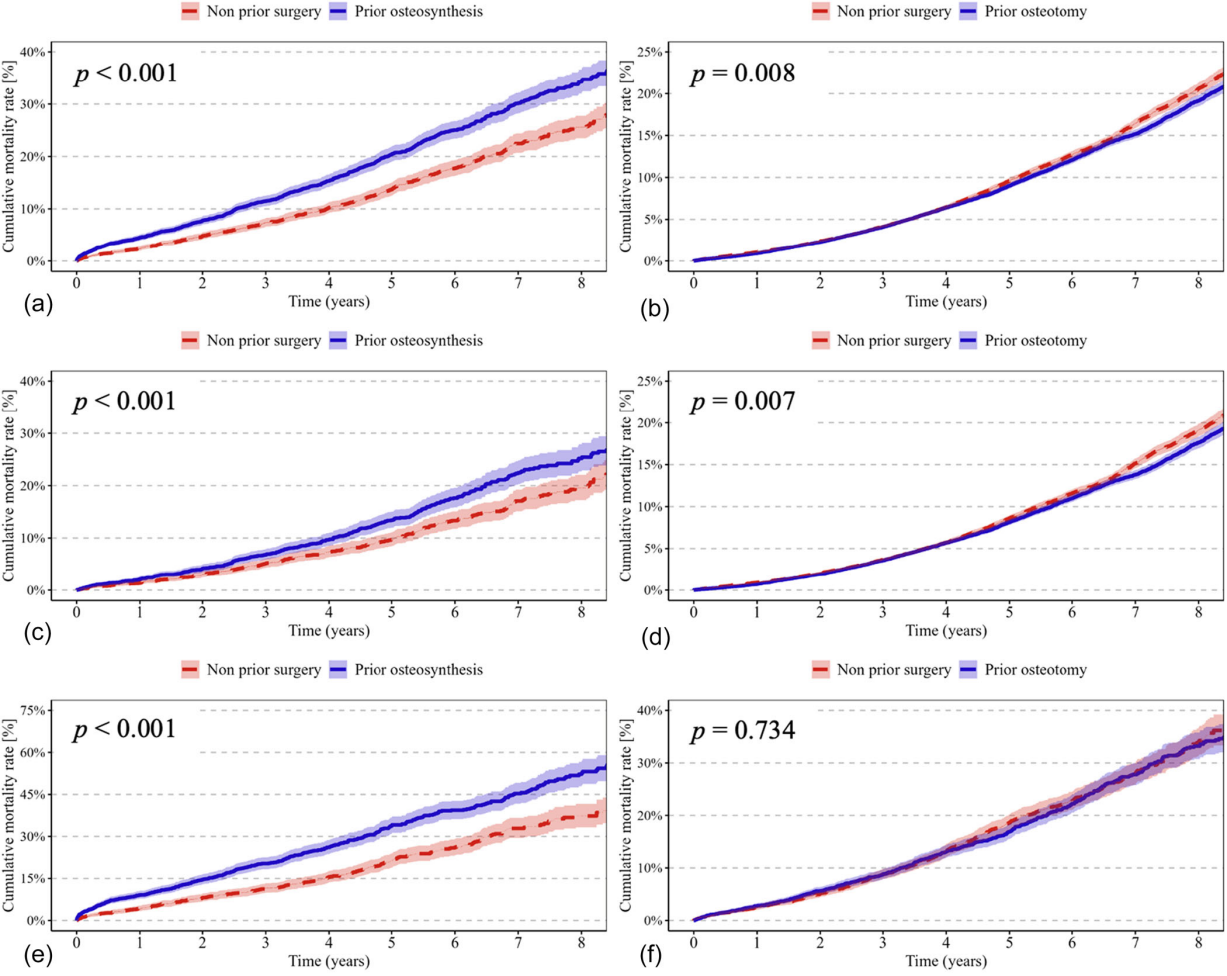
All‐cause mortality after total knee arthroplasty (TKA) (Kaplan–Meier; follow‐up ≤ 8 years). Comparison: prior osteosynthesis or osteotomy versus matched controls. Panels (letters): a and b = all TKA (a, osteosynthesis; b, osteotomy); c and d = unconstrained (c, osteosynthesis; d, osteotomy); e and f = constrained (e, osteosynthesis; f, osteotomy). Shaded areas:95% CI; *p*‐values: log‐rank. CI, confidence interval.

All‐cause mortality: A modest increase was observed after osteosynthesis, whereas osteotomy showed no overall increase (Figure 3; Tables 2 and 3).

### Secondary endpoints

**Figure 4:**
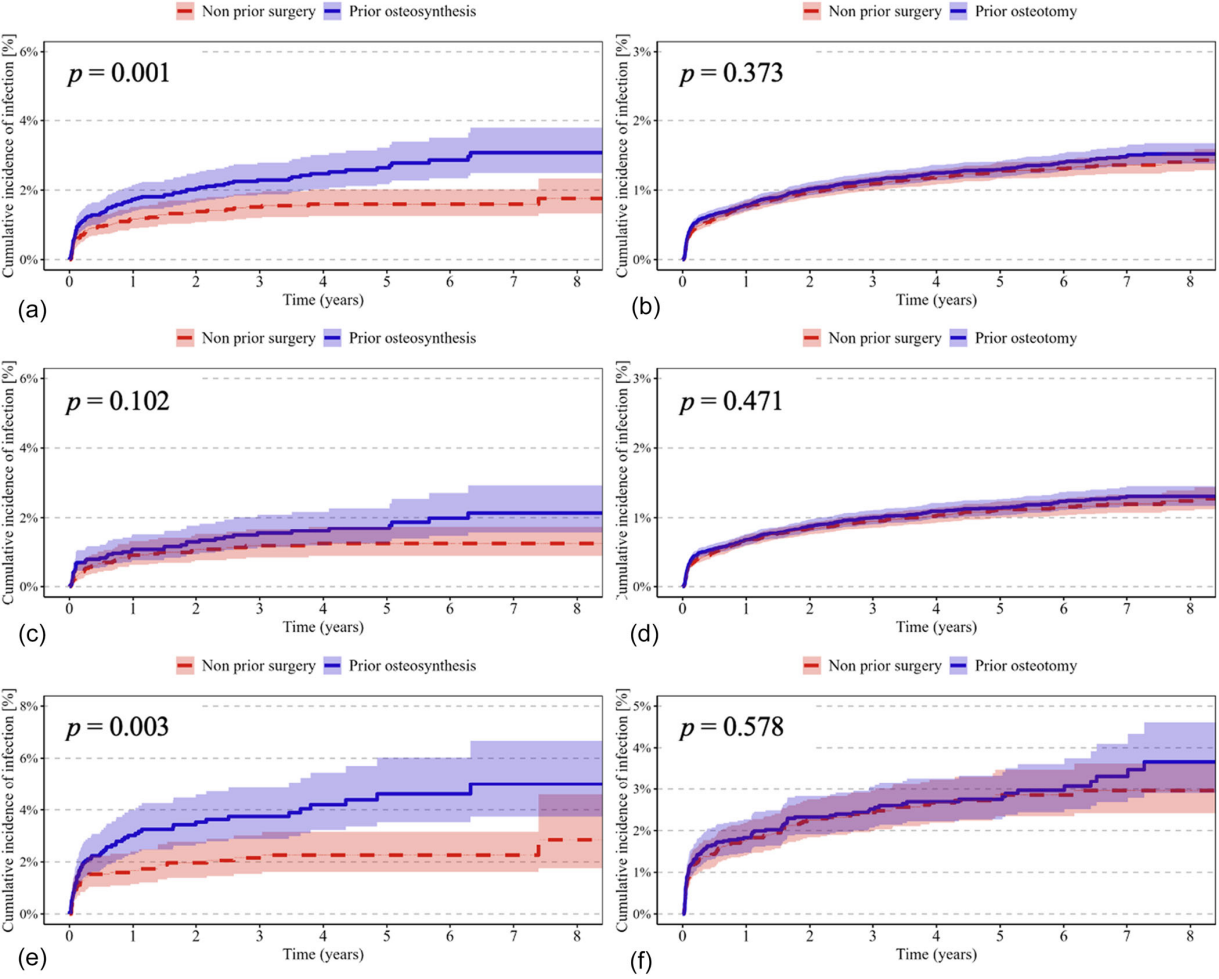
Postoperative infection after total knee arthroplasty (TKA) (Kaplan–Meier; follow‐up ≤ 8 years). Comparison: prior osteosynthesis or osteotomy versus matched controls. Panels: a and b = all TKA (a, osteosynthesis; b, osteotomy); c and d = unconstrained (c, osteosynthesis; d, osteotomy); e and f = constrained (e, osteosynthesis; f, osteotomy). Shaded areas: 95% CI; *p*‐values: log‐rank. CI, confidence interval.

After osteosynthesis, hazards were higher for infection, PPF and aseptic loosening (Table 2). After osteotomy, no significant differences were detected across age/comorbidity strata (Table 3). Figure 4 visualises the infection endpoint: after osteosynthesis, the Kaplan–Meier curve separates early and remains higher—including in constrained TKAs—whereas after osteotomy it closely tracks the control curve across strata. Kaplan–Meier curves for PPF and aseptic loosening are provided in the Supplement (Supporting Information S6: Figures 1 and 2).

## DISCUSSION

The most important finding of the present study was that prior osteosynthesis—but not osteotomy—was associated with an increased risk of revision after TKA, predominantly in conjunction with PJI, whereas outcomes after osteotomy were comparable to primary OA. In addition, a modest increase in all‐cause mortality was observed after osteosynthesis, while osteotomy showed no overall increase.

Osteosynthesis and osteotomy were distinguished using the OPS 2025 classification, and revision, mortality, infection, PPF and aseptic loosening were evaluated. Across endpoints, the direction of effects was consistent with the biological plausibility of the two exposure types: osteosynthesis reflects prior fracture care with potential contamination and soft‐tissue compromise [2, 12], while osteotomy typically preserves soft tissues and vascularity and is undertaken in biologically more favourable patients [6, 9]. Taken together, these features plausibly explain the higher infection‐driven revision burden after osteosynthesis and the absence of excess risk after osteotomy.

Multiple mechanisms may underlie the elevated risk after osteosynthesis. First, retained or previously removed hardware may harbour biofilm that is introduced at the time of TKA, facilitating early PJI [10]. Second, fracture‐related scarring and vascular impairment can prolong surgery and weaken local host defence [2, 12]. Third, intra‐articular fracture patterns often necessitate higher implant constraint and larger implant surfaces, which—together with longer operative times—may further increase infection risk [11]. By contrast, osteotomy patients generally present with preserved soft‐tissue envelopes, staged hardware removal and intact joint landmarks, aligning with the comparable revision and infection rates reported here [3, 5, 8, 13, 14].

Prior registry and cohort reports that described higher failure rates after post‐traumatic TKA without clearly identifying mechanisms are extended by the present findings [4]. Consistent with Baker et al., concurrent hardware removal appears undesirable when hardware lies within the surgical field [1]. For osteotomy, mixed signals in prior registries and meta‐analyses likely reflect differences in matching strategies, implant selection and technique heterogeneity; the present large matched analysis supports no relevant excess risk for revision or infection after osteotomy [3, 5, 8, 14].

In patients with a history of osteosynthesis, the index TKA should be planned as a risk‐augmented procedure: screen for occult infection when suspicion exists (including aspiration), favour staged over concurrent hardware removal if implants lie within the field and use the least constraint compatible with stability [1, 11]. Patients should be counselled that both revision and infection risks are higher than in primary OA, whereas patients after osteotomy can expect outcomes similar to primary TKA.

The strengths of this study include an OPS‐based, granular exposure definition, very large matched cohorts with near‐complete follow‐up and consistent results across constrained and unconstrained implants, enhancing external validity.

### Limitations

This retrospective registry analysis is subject to residual confounding despite matching and prespecified strata. Coding errors cannot be fully excluded. The registry lacks patient‐reported outcome measures (PROMs) and detailed intraoperative data (including the exact timing of hardware removal), precluding functional inference and fine‐grained operative risk attribution. Socio‐economic factors, rehabilitation setting and adherence to follow‐up are not captured and may influence infection and mortality estimates. Finally, residual selection effects related to referral patterns and surgeon preference cannot be fully excluded. Future work should link registry data to microbiology, rehabilitation and socio‐economic datasets to quantify these effects and test interventional strategies (e.g., staged hardware pathways and targeted prophylaxis).

### Clinical relevance

For day‐to‐day practice, these data support (1) targeted infection diagnostics and optimisation before TKA in patients with prior osteosynthesis, (2) staged hardware removal when implants lie within the surgical field and (3) restraint with implant constraint to limit operative time and surface area. After osteotomy, standard perioperative pathways appear appropriate.

### Conclusion

Prior osteosynthesis—unlike osteotomy—was associated with higher risks after primary TKA: increased all‐cause revision (HR 1.81, 95% CI 1.52–2.15), PJI (HR 1.62), PPF (HR 8.54) and aseptic loosening (HR 2.38), alongside a modest rise in all‐cause mortality (HR 1.48). In contrast, prior osteotomy showed no increase in revision or secondary complications and no overall mortality increase, with findings stable across age/comorbidity strata; for revision and infection, no excess risk was observed in either constrained or unconstrained implants.

## AUTHOR CONTRIBUTIONS

**Nele Wagener**: Conceptualization; data curation; formal analysis; funding acquisition; investigation; methodology; project administration; resources; software; supervision; validation; visualization; writing—original draft; writing—review and editing. **Yinan Wu**: Data curation; formal analysis; writing—review and editing. **Alexander Grimberg**: Data curation; formal analysis; writing—review and editing. **Christian Hipfl**: Data curation; formal analysis; investigation; methodology; project administration; resources; software; supervision; validation; visualization; writing—review and editing. **Sebastian Hardt**: Conceptualization; data curation; formal analysis; funding acquisition; investigation; methodology; project administration; resources; software; supervision; validation; visualization; writing—original draft; writing—review and editing.

## CONFLICT OF INTEREST STATEMENT

The authors declare no conflicts of interest.

## ETHICS STATEMENT

Ethical approval was obtained from Kiel University (D 473/11).

## Supporting information

Supporting Information.

Supporting Information.

Supporting Information.

Supporting Information.

Supporting Information.

Supporting Information.

## Data Availability

Data available on request from the authors.
