# Supplementary material for: Prior osteosynthesis—unlike osteotomy—raises revision risk after total knee arthroplasty, predominantly via periprosthetic infection
Source: Knee Surg Sports Traumatol Arthrosc. 2025 Oct 28;34(8):2833–41. doi: 10.1002/ksa.70153 (PMC13418327; doi:10.1002/ksa.70153)
Supplement: Supplementary file 1 — Supporting Information. [file KSA-34-2833-s001.pdf]

ICD-10 Code	Krankheit (ICD-10-GM Bezeichnung)
M21.06	Valgusdeformität: Unterschenkel (X-Bein)
M21.16	Varusdeformität: Unterschenkel (O-Bein)
M21.26	Flexionsdeformität: Unterschenkel (Knie)
M17.-	Gonarthrose [Arthrose des Kniegelenkes]
M22.0	Habituelle Luxation der Patella
M22.1	Habituelle Subluxation der Patella
M22.2	Krankheiten im Patellofemoralbereich
M22.4	Chondromalacia patellae
M21.16	Varusdeformität (Bow-Leg)
M21.06	Valgusdeformität Unterschenkel (Knie)
M17.1	Sonstige primäre Gonarthrose
M17.2	Posttraumatische Gonarthrose, beidseitig
M17.3	Sonstige posttraumatische Gonarthrose
M17.4	Sonstige sekundäre Gonarthrose, beidseitig
M17.5	Sonstige sekundäre Gonarthrose
M17.9	Gonarthrose, nicht näher bezeichnet
M21.05	Valgusdeformität, anderenorts nicht klassifiziert: Beckenregion und Oberschenkel (distales Femur)
M21.06	Valgusdeformität, anderenorts nicht klassifiziert: Unterschenkel (proximale Tibia/Knie)
M21.15	Varusdeformität, anderenorts nicht klassifiziert: Beckenregion und Oberschenkel (distales Femur)
M21.16	Varusdeformität, anderenorts nicht klassifiziert: Unterschenkel (proximale Tibia/Knie)
M21.26	Flexionsdeformität: Knie (Beugefehlstellung)
M21.85	Sonstige näher bezeichnete erworbene Deformitäten: Beckenregion und Oberschenkel (z. B. Femurtorsion/-verbiegung)
M21.86	Sonstige näher bezeichnete erworbene Deformitäten: Unterschenkel (z. B. Genu recurvatum, Tibiatorsion)
M84.05	Frakturheilung in Fehlstellung: Beckenregion und Oberschenkel (inkl. distales Femur)
M84.06	Frakturheilung in Fehlstellung: Unterschenkel (inkl. proximale Tibia/Knie)
M93.2	Osteochondritis dissecans
M87.05	Idiopathische aseptische Knochennekrose: Beckenregion und Oberschenkel
M87.06	Idiopathische aseptische Knochennekrose: Unterschenkel
M87.15	Knochennekrose durch Arzneimittel: Beckenregion und Oberschenkel
M87.16	Knochennekrose durch Arzneimittel: Unterschenkel
M87.25	Knochennekrose durch vorangegangenes Trauma: Beckenregion und Oberschenkel
M87.26	Knochennekrose durch vorangegangenes Trauma: Unterschenkel
M87.35	Sonstige sekundäre Knochennekrose: Beckenregion und Oberschenkel
M87.36	Sonstige sekundäre Knochennekrose: Unterschenkel
M87.85	Sonstige Knochennekrose: Beckenregion und Oberschenkel
M87.86	Sonstige Knochennekrose: Unterschenkel
M87.95	Knochennekrose, nicht näher bezeichnet: Beckenregion und Oberschenkel
M87.96	Knochennekrose, nicht näher bezeichnet: Unterschenkel
M23.5	Chronische Instabilität des Kniegelenkes
M24.56	Gelenkkontraktur: Unterschenkel (Knie)
M24.66	Ankylose eines Gelenkes: Unterschenkel (Knie)

M21.75	Unterschiedliche Extremitätenlänge (erworben): Beckenregion und Oberschenkel (Femur)
M21.76	Unterschiedliche Extremitätenlänge (erworben): Unterschenkel (Tibia/Fibula)
M92.5	Juvenile Osteochondrose der Tibia und Fibula (z. B. Tibia vara)
Q68.2	Angeborene Deformität des Knies (z. B. Genu recurvatum, kongenitale Knieluxation)
Q74.1	Angeborene Fehlbildung des Knies (z. B. Patelladysplasie/-luxation, Genu valgum/varum)
