# Supplementary material for: Prior osteosynthesis—unlike osteotomy—raises revision risk after total knee arthroplasty, predominantly via periprosthetic infection
Source: Knee Surg Sports Traumatol Arthrosc. 2025 Oct 28;34(8):2833–41. doi: 10.1002/ksa.70153 (PMC13418327; doi:10.1002/ksa.70153)
Supplement: Supplementary file 2 — Supporting Information. [file KSA-34-2833-s002.pdf]

ICD-10-GM Code	Diagnose (DE)
S72.40	Distale Fraktur des Femurs: Teil nicht näher bezeichnet
S72.41	Distale Fraktur des Femurs: Condylus (lateralis) (medialis)
S72.42	Distale Fraktur des Femurs: Epiphyse, Epiphysenlösung
S72.43	Distale Fraktur des Femurs: Suprakondylär
S72.44	Distale Fraktur des Femurs: Interkondylär
S82.0	Fraktur der Patella
S82.11	Fraktur des proximalen Endes der Tibia: Mit Fraktur der Fibula (jeder Teil)
S82.18	Fraktur des proximalen Endes der Tibia: Sonstige
S82.41	Fraktur der Fibula, isoliert: Proximales Ende
M84.05	Frakturheilung in Fehlstellung: Beckenregion und Oberschenkel (inkl. distaler Femur)
M84.06	Frakturheilung in Fehlstellung: Unterschenkel (inkl. proximale Tibia/Fibula)
M84.15	Nichtvereinigung der Frakturenden [Pseudarthrose]: Beckenregion und Oberschenkel
M84.16	Nichtvereinigung der Frakturenden [Pseudarthrose]: Unterschenkel
M84.25	Verzögerte Frakturheilung: Beckenregion und Oberschenkel
M84.26	Verzögerte Frakturheilung: Unterschenkel
M84.45	Pathologische Fraktur, anderenorts nicht klassifiziert: Beckenregion und Oberschenkel
M84.46	Pathologische Fraktur, anderenorts nicht klassifiziert: Unterschenkel
M96.6	Knochenfraktur nach Einsetzen oder Entfernen eines orthopädischen Implantates, einer Gelenkprothese oder einer Knochenplatte
