# Supplementary material for: Prior osteosynthesis—unlike osteotomy—raises revision risk after total knee arthroplasty, predominantly via periprosthetic infection
Source: Knee Surg Sports Traumatol Arthrosc. 2025 Oct 28;34(8):2833–41. doi: 10.1002/ksa.70153 (PMC13418327; doi:10.1002/ksa.70153)
Supplement: Supplementary file 3 — Supporting Information. [file KSA-34-2833-s006.pdf]

Distal femur osteosynthesis

5-793.5h	Offene Reposition einer Fraktur am distalen Femur durch dynamische Kompressionsschraube
5-793.bh	Offene Reposition einer Fraktur am distalen Femur durch Marknagel
5-793.4h	Offene Reposition einer Fraktur am distalen Femur durch Winkelplatte
5-793.ah	Offene Reposition einer Fraktur am distalen Femur durch Marknagel mit Gelenkkomponente
5-793.ch	Offene Reposition einer einfachen Fraktur im Gelenkbereich eines langen Röhrenknochens: Durch Transfixationsnagel: Femur distal
5-793.eh	Offene Reposition einer einfachen Fraktur im Gelenkbereich eines langen Röhrenknochens: Durch (Blount-)Klammern: Femur distal
5-793.gh	Offene Reposition einer Fraktur am distalen Femur durch ESIN
5-793.kh	Offene Reposition einer Fraktur am distalen Femur durch winkelstabile Platte
5-793.mh	Offene Reposition einer einfachen Fraktur im Gelenkbereich eines langen Röhrenknochens: Durch Ringfixateur: Femur distal
5-793.nh	Offene Reposition einer einfachen Fraktur im Gelenkbereich eines langen Röhrenknochens: Durch Bewegungsfixateur: Femur distal
5-790.6h	Geschlossene Reposition einer Fraktur am distalen Femur durch Fixateur externe
5-790.ch	Geschlossene Reposition einer Fraktur am distalen Femur durch Transfixationsnagel
5-790.ph	Geschlossene Reposition einer Fraktur am distalen Femur durch Bewegungsfixateur
5-790.1h	Geschlossene Reposition mit Bohrdrahtosteosynthese am distalen Femur
5-794.3h	Offene Reposition einer Mehrfragment-Fraktur am distalen Femur durch Winkelplatte
5-794.kh	Offene Reposition einer Mehrfragment-Fraktur am distalen Femur durch winkelstabile Platte
5-794.nh	Offene Reposition einer Mehrfragment-Fraktur im Gelenkbereich eines langen Röhrenknochens: Durch Bewegungsfixateur: Femur distal
5-793.6h	Offene Reposition einer einfachen Fraktur im Gelenkbereich eines langen Röhrenknochens: Durch Fixateur externe: Femur distal
5-793.1h	Offene Reposition einer einfachen Fraktur im Gelenkbereich eines langen Röhrenknochens: Durch Schraube: Femur distal
5-793.3h	Offene Reposition einer einfachen Fraktur im Gelenkbereich eines langen Röhrenknochens: Durch Platte: Femur distal
5-793.2h	Offene Reposition einer einfachen Fraktur im Gelenkbereich eines langen Röhrenknochens: Durch Draht oder Zuggurtung/Cerclage: Femur distal
5-790.0h	Geschlossene Reposition bei Apophyseolyse mit Schraubenosteosynthese am distalen Femur
5-790.1h	Geschlossene Reposition einer Fraktur oder Epiphysenlösung mit Osteosynthese: Durch Draht oder Zuggurtung/Cerclage: Femur distal
5-790.2h	Geschlossene Reposition einer Fraktur, Luxation oder Epiphysenlösung mit Osteosynthese am distalen Femur durch Endernagel
5-790.xh	Geschlossene Reposition einer Fraktur oder Epiphysenlösung mit Osteosynthese: Sonstige: Femur distal
5-790.4h	Geschlossene Reposition einer Fraktur oder Epiphysenlösung mit Osteosynthese: Durch Verriegelungsnagel: Femur distal
5-790.5h	Geschlossene Reposition einer Fraktur oder Epiphysenlösung mit Osteosynthese: Durch Marknagel mit Gelenkkomponente: Femur distal
5-790.8h	Geschlossene Reposition einer Fraktur oder Epiphysenlösung mit Osteosynthese: Durch dynamische Kompressionsschraube: Femur distal
5-790.kh	Geschlossene Reposition einer Fraktur oder Epiphysenlösung mit Osteosynthese: Durch winkelstabile Platte: Femur distal
5-790.mh	Geschlossene Reposition einer Fraktur oder Epiphysenlösung mit Osteosynthese: Durch Ringfixateur: Femur distal
5-790.3h	Geschlossene Reposition einer Fraktur oder Epiphysenlösung mit Osteosynthese: Durch Marknagel mit Aufbohren der Markhöhle: Femur distal
5-790.7h	Geschlossene Reposition einer Fraktur oder Epiphysenlösung mit Osteosynthese: Durch Winkelplatte/Kondylenplatte: Femur distal
5-794.0h	Offene Reposition einer Mehrfragment-Fraktur im Gelenkbereich eines langen Röhrenknochens: Durch Schraube: Femur distal
5-794.bh	Offene Reposition einer Mehrfragment-Fraktur im Gelenkbereich eines langen Röhrenknochens: Durch Marknagel: Femur distal
5-794.xh	Offene Reposition einer Mehrfragment-Fraktur im Gelenkbereich eines langen Röhrenknochens: Sonstige: Femur distal
5-794.1h	Offene Reposition einer Mehrfragment-Fraktur im Gelenkbereich eines langen Röhrenknochens: Durch Draht oder Zuggurtung/Cerclage: Femur distal
5-794.2h	Offene Reposition einer Mehrfragment-Fraktur im Gelenkbereich eines langen Röhrenknochens: Durch Platte: Femur distal
5-794.4h	Offene Reposition einer Mehrfragment-Fraktur im Gelenkbereich eines langen Röhrenknochens: Durch dynamische Kompressionsschraube: Femur distal
5-794.5h	Offene Reposition einer Mehrfragment-Fraktur im Gelenkbereich eines langen Röhrenknochens: Durch Fixateur externe: Femur distal
5-794.ah	Offene Reposition einer Mehrfragment-Fraktur im Gelenkbereich eines langen Röhrenknochens: Durch Marknagel mit Gelenkkomponente: Femur distal
5-794.ch	Offene Reposition einer Mehrfragment-Fraktur im Gelenkbereich eines langen Röhrenknochens: Durch Transfixationsnagel: Femur distal
5-794.eh	Offene Reposition einer Mehrfragment-Fraktur im Gelenkbereich eines langen Röhrenknochens: Durch (Blount-)Klammern: Femur distal
5-794.gh	Offene Reposition einer Mehrfragment-Fraktur im Gelenkbereich eines langen Röhrenknochens: Durch intramedullären Draht: Femur distal
5-794.mh	Offene Reposition einer Mehrfragment-Fraktur im Gelenkbereich eines langen Röhrenknochens: Durch Ringfixateur: Femur distal
5-789.3h	Andere Operationen am Knochen: Revision von Osteosynthesematerial ohne Materialwechsel: Femur distal
5-78a.0h	Revision von Osteosynthesematerial mit Reosteosynthese: Durch Schraube: Femur distal
5-78a.2h	Revision von Osteosynthesematerial mit Reosteosynthese: Durch Platte: Femur distal
5-78a.4h	Revision von Osteosynthesematerial mit Reosteosynthese: Durch dynamische Kompressionsschraube: Femur distal
5-78a.6h	Revision von Osteosynthesematerial mit Reosteosynthese: Durch Marknagel: Femur distal
5-78a.7h	Revision von Osteosynthesematerial mit Reosteosynthese: Durch Verriegelungsnagel: Femur distal
5-78a.xh	Revision von Osteosynthesematerial mit Reosteosynthese: Sonstige: Femur distal
5-78a.5h	Revision von Osteosynthesematerial mit Reosteosynthese: Durch Marknagel mit Gelenkkomponente: Femur distal
5-78a.8h	Revision von Osteosynthesematerial mit Reosteosynthese: Durch Fixateur externe: Femur distal
5-78a.ch	Revision von Osteosynthesematerial mit Reosteosynthese: Durch Transfixationsnagel: Femur distal
5-78a.eh	Revision von Osteosynthesematerial mit Reosteosynthese: Durch (Blount-)Klammern: Femur distal
5-78a.gh	Revision von Osteosynthesematerial mit Reosteosynthese: Durch intramedullären Draht: Femur distal
5-78a.kh	Revision von Osteosynthesematerial mit Reosteosynthese: Durch winkelstabile Platte: Femur distal
5-78a.mh	Revision von Osteosynthesematerial mit Reosteosynthese: Durch Ringfixateur: Femur distal
5-78a.nh	Revision von Osteosynthesematerial mit Reosteosynthese: Durch Bewegungsfixateur: Femur distal
5-78a.3h	Revision von Osteosynthesematerial mit Reosteosynthese: Durch Winkelplatte/Kondylenplatte: Femur distal
5-78a.1h	Revision von Osteosynthesematerial mit Reosteosynthese: Durch Draht oder Zuggurtung/Cerclage: Femur distal
5-787.5h	Entfernung von Osteosynthesematerial: Dynamische Kompressionsschraube: Femur distal
5-787.6h	Entfernung von Osteosynthesematerial: Marknagel: Femur distal
5-787.8h	Entfernung von Osteosynthesematerial: Verriegelungsnagel: Femur distal
5-787.4h	Entfernung einer Winkelplatte aus dem distalen Femur
5-787.7h	Entfernung von Osteosynthesematerial: Marknagel mit Gelenkkomponente: Femur distal
5-787.kh	Entfernung von Osteosynthesematerial: Winkelstabile Platte: Femur distal
5-787.mh	Entfernung von Osteosynthesematerial: Ringfixateur: Femur distal
5-787.0h	Entfernung einer Draht-Schlinge aus dem distalen Femur
5-787.9h	Entfernung von Osteosynthesematerial: Fixateur externe: Femur distal
5-787.ch	Entfernung von Osteosynthesematerial: Transfixationsnagel: Femur distal
5-787.eh	Entfernung von Klammern aus dem distalen Femur
5-787.2h	Entfernung von Osteosynthesematerial: Zuggurtung/Cerclage: Femur distal
5-787.xh	Entfernung von Osteosynthesematerial: Sonstige: Femur distal
5-787.j0	Entfernung eines Teleskopnagels aus einem Knochen

Patella osteosynthesis

5-794.1j Zuggurtungsosteosynthese einer Mehrfragment-Fraktur an der Patella

5-790.1j	Geschlossene Reposition mit Bohrdrahtosteosynthese an der Patella
5-794.kj	Offene Reposition einer Mehrfragment-Fraktur im Gelenkbereich eines langen Röhrenknochens: Durch winkelstabile Platte: Patella
5-794.nj	Offene Reposition einer Mehrfragment-Fraktur im Gelenkbereich eines langen Röhrenknochens: Durch Bewegungsfixateur: Patella
5-793.ej	Offene Reposition einer einfachen Fraktur im Gelenkbereich eines langen Röhrenknochens: Durch (Blount-)Klammern: Patella
5-793.kj	Offene Reposition einer einfachen Fraktur im Gelenkbereich eines langen Röhrenknochens: Durch winkelstabile Platte: Patella
5-793.6j	Offene Reposition einer einfachen Fraktur im Gelenkbereich eines langen Röhrenknochens: Durch Fixateur externe: Patella
5-790.0j	Geschlossene Reposition bei Apophyseolyse mit Schraubenosteosynthese an der Patella
5-790.pj	Geschlossene Reposition einer Fraktur oder Epiphysenlösung mit Osteosynthese: Durch Bewegungsfixateur: Patella
5-794.1j	Offene Reposition einer Mehrfragment-Fraktur im Gelenkbereich eines langen Röhrenknochens: Durch Draht oder Zuggurtung/Cerclage: Patella
5-794.0j	Offene Reposition einer Mehrfragment-Fraktur im Gelenkbereich eines langen Röhrenknochens: Durch Schraube: Patella
5-794.xj	Offene Reposition einer Mehrfragment-Fraktur im Gelenkbereich eines langen Röhrenknochens: Sonstige: Patella
5-794.2j	Offene Reposition einer Mehrfragment-Fraktur im Gelenkbereich eines langen Röhrenknochens: Durch Platte: Patella
5-794.5j	Offene Reposition einer Mehrfragment-Fraktur im Gelenkbereich eines langen Röhrenknochens: Durch Fixateur externe: Patella
5-794.ej	Offene Reposition einer Mehrfragment-Fraktur im Gelenkbereich eines langen Röhrenknochens: Durch (Blount-)Klammern: Patella
5-790.6j	Geschlossene Reposition einer Fraktur an der Patella durch Fixateur externe
5-790.1j	Geschlossene Reposition einer Fraktur oder Epiphysenlösung mit Osteosynthese: Durch Draht oder Zuggurtung/Cerclage: Patella
5-790.xj	Geschlossene Reposition einer Fraktur oder Epiphysenlösung mit Osteosynthese: Sonstige: Patella
5-790.kj	Geschlossene Reposition einer Fraktur oder Epiphysenlösung mit Osteosynthese: Durch winkelstabile Platte: Patella
5-793.nj	Offene Reposition einer einfachen Fraktur im Gelenkbereich eines langen Röhrenknochens: Durch Bewegungsfixateur: Patella
5-793.1j	Offene Reposition einer einfachen Fraktur im Gelenkbereich eines langen Röhrenknochens: Durch Schraube: Patella
5-793.2j	Offene Reposition einer einfachen Fraktur im Gelenkbereich eines langen Röhrenknochens: Durch Draht oder Zuggurtung/Cerclage: Patella
5-793.3j	Offene Reposition einer einfachen Fraktur im Gelenkbereich eines langen Röhrenknochens: Durch Platte: Patella
5-793.3k	Offene Reposition einer einfachen Fraktur im Gelenkbereich eines langen Röhrenknochens: Durch Platte: Tibia proximal
5-793.3n	Offene Reposition einer einfachen Fraktur im Gelenkbereich eines langen Röhrenknochens: Durch Platte: Tibia distal
5-793.3p	Offene Reposition einer einfachen Fraktur im Gelenkbereich eines langen Röhrenknochens: Durch Platte: Fibula proximal
5-793.3r	Offene Reposition einer einfachen Fraktur im Gelenkbereich eines langen Röhrenknochens: Durch Platte: Fibula distal
5-789.3j	Andere Operationen am Knochen: Revision von Osteosynthesematerial ohne Materialwechsel: Patella
5-78a.0j	Revision von Osteosynthesematerial mit Reosteosynthese: Durch Schraube: Patella
5-78a.2j	Revision von Osteosynthesematerial mit Reosteosynthese: Durch Platte: Patella
5-78a.kj	Revision von Osteosynthesematerial mit Reosteosynthese: Durch winkelstabile Platte: Patella
5-78a.nj	Revision von Osteosynthesematerial mit Reosteosynthese: Durch Bewegungsfixateur: Patella
5-78a.xj	Revision von Osteosynthesematerial mit Reosteosynthese: Sonstige: Patella
5-78a.8j	Revision von Osteosynthesematerial mit Reosteosynthese: Durch Fixateur externe: Patella
5-78a.ej	Revision von Osteosynthesematerial mit Reosteosynthese: Durch (Blount-)Klammern: Patella
5-78a.1j	Revision von Osteosynthesematerial mit Reosteosynthese: Durch Draht oder Zuggurtung/Cerclage: Patella
5-787.kj	Entfernung von Osteosynthesematerial: Winkelstabile Platte: Patella
5-787.9j	Entfernung eines Ilizarov-Fixateurs aus der Patella
5-787.0j	Entfernung einer Draht-Schlinge aus der Patella
5-787.ej	Entfernung von Klammern aus der Patella
5-787.2j	Entfernung von Osteosynthesematerial: Zuggurtung/Cerclage: Patella
5-787.j0	Entfernung eines Teleskopnagels aus einem Knochen
5-787.xj	Entfernung von Osteosynthesematerial: Sonstige: Patella
5-793.2j	Zuggurtungsosteosynthese einer einfachen Patellafraktur

Proximal tibia osteosynthesis

5-793.5k	Offene Reposition einer einfachen Fraktur im Gelenkbereich eines langen Röhrenknochens: Durch dynamische Kompressionsschraube: Tibia proximal
5-793.ak	Offene Reposition einer einfachen Fraktur im Gelenkbereich eines langen Röhrenknochens: Durch Marknagel mit Gelenkkomponente: Tibia proximal
5-793.bk	Offene Reposition einer einfachen Fraktur im Gelenkbereich eines langen Röhrenknochens: Durch Marknagel: Tibia proximal
5-793.ck	Offene Reposition einer einfachen Fraktur im Gelenkbereich eines langen Röhrenknochens: Durch Transfixationsnagel: Tibia proximal
5-793.ek	Offene Reposition einer einfachen Fraktur im Gelenkbereich eines langen Röhrenknochens: Durch (Blount-)Klammern: Tibia proximal
5-793.kk	Offene Reposition einer einfachen Fraktur im Gelenkbereich eines langen Röhrenknochens: Durch winkelstabile Platte: Tibia proximal
5-793.mk	Offene Reposition einer einfachen Fraktur im Gelenkbereich eines langen Röhrenknochens: Durch Ringfixateur: Tibia proximal
5-793.nk	Offene Reposition einer einfachen Fraktur im Gelenkbereich eines langen Röhrenknochens: Durch Bewegungsfixateur: Tibia proximal
5-794.3k	Offene Reposition einer Mehrfragment-Fraktur an der proximalen Tibia durch Winkelplatte
5-794.kk	Offene Reposition einer Mehrfragment-Fraktur an der proximalen Tibia durch winkelstabile Platte
5-794.nk	Offene Reposition einer Mehrfragment-Fraktur im Gelenkbereich eines langen Röhrenknochens: Durch Bewegungsfixateur: Tibia proximal
5-790.1k	Geschlossene Reposition mit Bohrdrahtosteosynthese an der proximalen Tibia
5-793.4k	Offene Reposition einer einfachen Fraktur im Gelenkbereich eines langen Röhrenknochens: Durch Winkelplatte/Kondylenplatte: Tibia proximal
5-793.6k	Offene Reposition einer einfachen Fraktur im Gelenkbereich eines langen Röhrenknochens: Durch Fixateur externe: Tibia proximal
5-793.1k	Offene Reposition einer einfachen Fraktur im Gelenkbereich eines langen Röhrenknochens: Durch Schraube: Tibia proximal
5-793.2k	Offene Reposition einer einfachen Fraktur im Gelenkbereich eines langen Röhrenknochens: Durch Draht oder Zuggurtung/Cerclage: Tibia proximal
5-793.3k	Offene Reposition einer einfachen Fraktur im Gelenkbereich eines langen Röhrenknochens: Durch Platte: Tibia proximal
5-793.3n	Offene Reposition einer einfachen Fraktur im Gelenkbereich eines langen Röhrenknochens: Durch Platte: Tibia distal
5-793.3p	Offene Reposition einer einfachen Fraktur im Gelenkbereich eines langen Röhrenknochens: Durch Platte: Fibula proximal
5-793.3r	Offene Reposition einer einfachen Fraktur im Gelenkbereich eines langen Röhrenknochens: Durch Platte: Fibula distal
5-794.0k	Offene Reposition einer Mehrfragment-Fraktur im Gelenkbereich eines langen Röhrenknochens: Durch Schraube: Tibia proximal
5-794.2k	Offene Reposition einer Mehrfragment-Fraktur im Gelenkbereich eines langen Röhrenknochens: Durch Platte: Tibia proximal
5-794.4k	Offene Reposition einer Mehrfragment-Fraktur im Gelenkbereich eines langen Röhrenknochens: Durch dynamische Kompressionsschraube: Tibia proximal
5-794.bk	Offene Reposition einer Mehrfragment-Fraktur im Gelenkbereich eines langen Röhrenknochens: Durch Marknagel: Tibia proximal
5-794.gk	Offene Reposition einer Mehrfragment-Fraktur im Gelenkbereich eines langen Röhrenknochens: Durch intramedullären Draht: Tibia proximal
5-794.xk	Offene Reposition einer Mehrfragment-Fraktur im Gelenkbereich eines langen Röhrenknochens: Sonstige: Tibia proximal
5-794.1k	Offene Reposition einer Mehrfragment-Fraktur im Gelenkbereich eines langen Röhrenknochens: Durch Draht oder Zuggurtung/Cerclage: Tibia proximal
5-794.5k	Offene Reposition einer Mehrfragment-Fraktur im Gelenkbereich eines langen Röhrenknochens: Durch Fixateur externe: Tibia proximal
5-794.ak	Offene Reposition einer Mehrfragment-Fraktur im Gelenkbereich eines langen Röhrenknochens: Durch Marknagel mit Gelenkkomponente: Tibia proximal
5-794.ck	Offene Reposition einer Mehrfragment-Fraktur im Gelenkbereich eines langen Röhrenknochens: Durch Transfixationsnagel: Tibia proximal
5-794.ek	Offene Reposition einer Mehrfragment-Fraktur im Gelenkbereich eines langen Röhrenknochens: Durch (Blount-)Klammern: Tibia proximal
5-794.mk	Offene Reposition einer Mehrfragment-Fraktur im Gelenkbereich eines langen Röhrenknochens: Durch Ringfixateur: Tibia proximal
5-790.0k	Geschlossene Reposition bei Apophyseolyse mit Schraubenosteosynthese an der proximalen Tibia
5-790.6k	Geschlossene Reposition einer Fraktur an der proximalen Tibia durch Fixateur externe
5-790.ck	Geschlossene Reposition einer Fraktur an der proximalen Tibia durch Transfixationsnagel
5-790.pk	Geschlossene Reposition einer Fraktur an der proximalen Tibia durch Bewegungsfixateur
5-790.1k	Geschlossene Reposition einer Fraktur oder Epiphysenlösung mit Osteosynthese: Durch Draht oder Zuggurtung/Cerclage: Tibia proximal
5-790.2k	Geschlossene Reposition einer Fraktur, Luxation oder Epiphysenlösung mit Osteosynthese an der proximalen Tibia durch Endernagel
5-790.xk	Geschlossene Reposition einer Fraktur oder Epiphysenlösung mit Osteosynthese: Sonstige: Tibia proximal
5-790.4k	Geschlossene Reposition einer Fraktur oder Epiphysenlösung mit Osteosynthese: Durch Verriegelungsnagel: Tibia proximal
5-790.5k	Geschlossene Reposition einer Fraktur oder Epiphysenlösung mit Osteosynthese: Durch Marknagel mit Gelenkkomponente: Tibia proximal
5-790.8k	Geschlossene Reposition einer Fraktur oder Epiphysenlösung mit Osteosynthese: Durch dynamische Kompressionsschraube: Tibia proximal
5-790.kk	Geschlossene Reposition einer Fraktur oder Epiphysenlösung mit Osteosynthese: Durch winkelstabile Platte: Tibia proximal
5-790.mk	Geschlossene Reposition einer Fraktur oder Epiphysenlösung mit Osteosynthese: Durch Ringfixateur: Tibia proximal
5-790.3k	Geschlossene Reposition einer Fraktur oder Epiphysenlösung mit Osteosynthese: Durch Marknagel mit Aufbohren der Markhöhle: Tibia proximal
5-790.7k	Geschlossene Reposition einer Fraktur oder Epiphysenlösung mit Osteosynthese: Durch Winkelplatte/Kondylenplatte: Tibia proximal
5-79a.xj	Geschlossene Reposition einer Gelenkluxation mit Osteosynthese: Sonstige: Proximales Tibiofibulargelenk
5-79a.1j	Geschlossene Reposition einer Gelenkluxation mit Osteosynthese: Durch Draht oder Zuggurtung/Cerclage: Proximales Tibiofibulargelenk
5-79b.xj	Offene Reposition einer Gelenkluxation: Sonstige: Proximales Tibiofibulargelenk
5-79b.1j	Offene Reposition einer Gelenkluxation: Durch Draht oder Zuggurtung/Cerclage: Proximales Tibiofibulargelenk
5-789.3k	Andere Operationen am Knochen: Revision von Osteosynthesematerial ohne Materialwechsel: Tibia proximal
5-78a.0k	Revision von Osteosynthesematerial mit Reosteosynthese: Durch Schraube: Tibia proximal
5-78a.2k	Revision von Osteosynthesematerial mit Reosteosynthese: Durch Platte: Tibia proximal
5-78a.4k	Revision von Osteosynthesematerial mit Reosteosynthese: Durch dynamische Kompressionsschraube: Tibia proximal
5-78a.6k	Revision von Osteosynthesematerial mit Reosteosynthese: Durch Marknagel: Tibia proximal
5-78a.7k	Revision von Osteosynthesematerial mit Reosteosynthese: Durch Verriegelungsnagel: Tibia proximal
5-78a.xk	Revision von Osteosynthesematerial mit Reosteosynthese: Sonstige: Tibia proximal
5-78a.3k	Revision von Osteosynthesematerial mit Reosteosynthese: Durch Winkelplatte/Kondylenplatte: Tibia proximal
5-78a.5k	Revision von Osteosynthesematerial mit Reosteosynthese: Durch Marknagel mit Gelenkkomponente: Tibia proximal
5-78a.8k	Revision von Osteosynthesematerial mit Reosteosynthese: Durch Fixateur externe: Tibia proximal
5-78a.ck	Revision von Osteosynthesematerial mit Reosteosynthese: Durch Transfixationsnagel: Tibia proximal
5-78a.ek	Revision von Osteosynthesematerial mit Reosteosynthese: Durch (Blount-)Klammern: Tibia proximal
5-78a.gk	Revision von Osteosynthesematerial mit Reosteosynthese: Durch intramedullären Draht: Tibia proximal
5-78a.kk	Revision von Osteosynthesematerial mit Reosteosynthese: Durch winkelstabile Platte: Tibia proximal
5-78a.mk	Revision von Osteosynthesematerial mit Reosteosynthese: Durch Ringfixateur: Tibia proximal
5-78a.nk	Revision von Osteosynthesematerial mit Reosteosynthese: Durch Bewegungsfixateur: Tibia proximal
5-78a.1k	Revision von Osteosynthesematerial mit Reosteosynthese: Durch Draht oder Zuggurtung/Cerclage: Tibia proximal
5-787.5k	Entfernung von Osteosynthesematerial: Dynamische Kompressionsschraube: Tibia proximal
5-787.6k	Entfernung von Osteosynthesematerial: Marknagel: Tibia proximal
5-787.8k	Entfernung von Osteosynthesematerial: Verriegelungsnagel: Tibia proximal
5-787.4k	Entfernung einer Winkelplatte aus der proximalen Tibia
5-787.7k	Entfernung von Osteosynthesematerial: Marknagel mit Gelenkkomponente: Tibia proximal
5-787.9k	Entfernung von Osteosynthesematerial: Fixateur externe: Tibia proximal
5-787.ck	Entfernung von Osteosynthesematerial: Transfixationsnagel: Tibia proximal
5-787.ek	Entfernung von Osteosynthesematerial: (Blount-)Klammern: Tibia proximal
5-787.kk	Entfernung von Osteosynthesematerial: Winkelstabile Platte: Tibia proximal
5-787.mk	Entfernung von Osteosynthesematerial: Ringfixateur: Tibia proximal
5-787.0k	Entfernung einer Draht-Schlinge aus der proximalen Tibia
5-787.xk	Entfernung von Osteosynthesematerial: Sonstige: Tibia proximal
5-787.2k	Entfernung von Osteosynthesematerial: Zuggurtung/Cerclage: Tibia proximal
5-787.j0	Entfernung eines Teleskopnagels aus einem Knochen
5-810.3j	Arthroskopische Gelenkoperation: Entfernung von Osteosynthesematerial: Proximales Tibiofibulargelenk
