# Supplementary material for: Prior osteosynthesis—unlike osteotomy—raises revision risk after total knee arthroplasty, predominantly via periprosthetic infection
Source: Knee Surg Sports Traumatol Arthrosc. 2025 Oct 28;34(8):2833–41. doi: 10.1002/ksa.70153 (PMC13418327; doi:10.1002/ksa.70153)
Supplement: Supplementary file 4 — Supporting Information. [file KSA-34-2833-s005.pdf]

Distal femur osteotomy

5-780.0h	Exploration am distalen Femur durch Inzision
5-781.8h	Osteotomie und Korrekturosteotomie: Komplexe (mehrdimensionale) Osteotomie: Femur distal
5-781.xh	Osteotomie und Korrekturosteotomie: Sonstige: Femur distal
5-780.7h	Inzision am Knochen, septisch und aseptisch: Sequesterotomie: Femur distal
5-780.8h	Inzision am Knochen, septisch und aseptisch: Sequesterotomie mit Einlegen eines Medikamententrägers: Femur distal
5-780.9h	Kombination einer anderen Knocheninzision am distalen Femur mit Einlegen eines Medikamententrägers
5-780.4h	Inzision am Knochen, septisch und aseptisch: Einlegen eines Medikamententrägers: Femur distal
5-781.9h	Osteotomie und Korrekturosteotomie: Komplexe (mehrdimensionale) Osteotomie mit Achsenkorrektur: Femur distal
5-781.ah	Osteotomie und Korrekturosteotomie: Osteotomie ohne Achsenkorrektur: Femur distal
5-780.2h	Saug-Spül-Drainage am distalen Femur
5-780.6h	Inzision am Knochen, septisch und aseptisch: Debridement: Femur distal
5-780.3h	Fremdkörperentfernung aus dem distalen Femur durch Inzision
5-780.5h	Inzision am Knochen, septisch und aseptisch: Entfernen eines Medikamententrägers: Femur distal
5-780.1h	Inzision am Knochen, septisch und aseptisch: Knochenbohrung: Femur distal
5-781.4h	Osteotomie und Korrekturosteotomie: Verlängerungsosteotomie: Femur distal
5-781.6h	Osteotomie und Korrekturosteotomie: Varisierende (De-)Rotationsosteotomie: Femur distal
5-781.0h	Osteotomie und Korrekturosteotomie: Valgisierende Osteotomie: Femur distal
5-781.1h	Osteotomie und Korrekturosteotomie: Varisierende Osteotomie: Femur distal
5-781.2h	Osteotomie und Korrekturosteotomie: (De-)Rotationsosteotomie: Femur distal
5-781.3h	Osteotomie und Korrekturosteotomie: Verkürzungsosteotomie: Femur distal
5-789.4h	Andere Operationen am Knochen: Therapeutische Epiphyseolyse: Femur distal
5-789.1h	Andere Operationen am Knochen: Epiphyseodese, temporär: Femur distal
5-789.2h	Andere Operationen am Knochen: Epiphyseodese, permanent: Femur distal

Osteotomy patella

5-780.0j	Exploration an der Patella durch Inzision
5-804.4	Offen chirurgische Operationen an der Patella und ihrem Halteapparat: Patellaosteotomie
5-804.4	Offene Keilosteotomie an der Patella
5-780.7j	Inzision am Knochen, septisch und aseptisch: Sequesterotomie: Patella
5-780.2j	Saug-Spül-Drainage an der Patella
5-780.8j	Inzision am Knochen, septisch und aseptisch: Sequesterotomie mit Einlegen eines Medikamententrägers: Patella
5-780.6j	Ausmuldung und Saug-Spül-Drainage bei Osteomyelitis an der Patella
5-780.3j	Fremdkörperentfernung aus der Patella durch Inzision
5-780.4j	Inzision am Knochen, septisch und aseptisch: Einlegen eines Medikamententrägers: Patella
5-780.5j	Inzision am Knochen, septisch und aseptisch: Entfernen eines Medikamententrägers: Patella
5-780.9j	Kombination einer anderen Knocheninzision an der Patella mit Einlegen eines Medikamententrägers
5-780.9j	Knocheninzision an der Patella mit Einlegen eines Medikamententrägers mit Saug-Spül-Drainage
5-780.1j	Inzision am Knochen, septisch und aseptisch: Knochenbohrung: Patella
5-780.1j	Knochenbohrung an der Patella durch septische und aseptische Inzision mit Saug-Spül-Drainage

Proximal tibia osteotomy

5-780.0k	Exploration an der proximalen Tibia durch Inzision
5-781.xk	Verschiebeosteotomie an der proximalen Tibia
5-781.8k	Osteotomie und Korrekturosteotomie: Komplexe (mehrdimensionale) Osteotomie: Tibia proximal
5-781.xk	Osteotomie und Korrekturosteotomie: Sonstige: Tibia proximal
5-781.0k	Osteotomie und Korrekturosteotomie: Valgisierende Osteotomie: Tibia proximal
5-780.7k	Sequesterotomie an der proximalen Tibia
5-781.9k	Osteotomie und Korrekturosteotomie: Komplexe (mehrdimensionale) Osteotomie mit Achsenkorrektur: Tibia proximal
5-781.ak	Osteotomie und Korrekturosteotomie: Osteotomie ohne Achsenkorrektur: Tibia proximal
5-780.8k	Inzision am Knochen, septisch und aseptisch: Sequesterotomie mit Einlegen eines Medikamententrägers: Tibia proximal
5-780.6k	Debridement an der proximalen Tibia
5-780.2k	Saug-Spül-Drainage an der proximalen Tibia
5-780.3k	Fremdkörperentfernung aus der proximalen Tibia durch Inzision
5-780.4k	Einlegen eines Medikamententrägers an der proximalen Tibia
5-780.5k	Entfernung eines Medikamententrägers an der proximalen Tibia
5-780.9k	Kombination einer anderen Knocheninzision an der proximalen Tibia mit Einlegen eines Medikamententrägers
5-780.1k	Inzision am Knochen, septisch und aseptisch: Knochenbohrung: Tibia proximal
5-781.4k	Osteotomie und Korrekturosteotomie: Verlängerungsosteotomie: Tibia proximal
5-781.6k	Osteotomie und Korrekturosteotomie: Varisierende (De-)Rotationsosteotomie: Tibia proximal
5-781.1k	Osteotomie und Korrekturosteotomie: Varisierende Osteotomie: Tibia proximal
5-781.2k	Osteotomie und Korrekturosteotomie: (De-)Rotationsosteotomie: Tibia proximal
5-781.3k	Osteotomie und Korrekturosteotomie: Verkürzungsosteotomie: Tibia proximal
5-781.5k	Osteotomie und Korrekturosteotomie: Valgisierende (De-)Rotationsosteotomie: Tibia proximal
5-789.4k	Andere Operationen am Knochen: Therapeutische Epiphyseolyse: Tibia proximal
5-789.1k	Andere Operationen am Knochen: Epiphyseodese, temporär: Tibia proximal
5-789.2k	Andere Operationen am Knochen: Epiphyseodese, permanent: Tibia proximal
