# Supplementary material for: Prior osteosynthesis—unlike osteotomy—raises revision risk after total knee arthroplasty, predominantly via periprosthetic infection
Source: Knee Surg Sports Traumatol Arthrosc. 2025 Oct 28;34(8):2833–41. doi: 10.1002/ksa.70153 (PMC13418327; doi:10.1002/ksa.70153)
Supplement: Supplementary file 5 — Supporting Information. [file KSA-34-2833-s004.docx]

**Supporting information**

**Power and sample size
Because an a priori hypothesis specified a higher revision hazard after prior osteosynthesis, a sample‑size justification for the log‑rank test was performed using the MedCalc “Sample size for Survival analysis” (**v22.0, MedCalc Software Ltd., Ostend, Belgium) **approach (two‑sided α = 0.05, 1−β = 0.80; 1:1 allocation). Using the 8‑year Kaplan–Meier revision‑free survival from the matched cohorts (control 0.944 vs prior osteosynthesis 0.905), the required sample size is ≈ 718 per group (≈ 1,436 total). For a 90 % power scenario, ≈ 960–961 per group (≈ 1,920 total) would be required. The matched osteosynthesis cohorts in this study (N = 5 039 per group) exceed these requirements by a wide margin, confirming adequate power.
For completeness, an events‑based Schoenfeld/Freedman check was added. With the target effect size set to the observed hazard ratio (HR = 1.81), the required number of events is D_req = 4·(Z_{1−α/2}+Z_{1−β})²/(ln HR)² ≈ 89 events (≈ 120 for 90 % power). Under the planning incidences derived from the 8‑year Kaplan–Meier curves (events ≈ 5.6 % vs 9.5 %; average ≈ 7.6 %), this corresponds to ≈ 1 180 total (≈ 590 per group). This criterion is likewise surpassed by the available cohorts.
For the large osteotomy comparison (N = 48,391 per group), a sensitivity analysis expresses precision as the minimal detectable hazard ratio (MDHR) at 80 % power across plausible 8‑year incidences. Assuming a cumulative incidence of 2 % / 3 % / 5 % across both arms, the MDHRs are ≈ 1.14 / 1.11 / 1.08, respectively, indicating high sensitivity to moderate relative differences.
All calculations assume proportional hazards, two‑sided testing and 1:1 allocation; results are equivalent for the log‑rank test and Cox models.**
