# Supplementary material for: Prior osteosynthesis—unlike osteotomy—raises revision risk after total knee arthroplasty, predominantly via periprosthetic infection
Source: Knee Surg Sports Traumatol Arthrosc. 2025 Oct 28;34(8):2833–41. doi: 10.1002/ksa.70153 (PMC13418327; doi:10.1002/ksa.70153)
Supplement: Supplementary file 6 — Supporting Information. [file KSA-34-2833-s003.docx]

**Supporting Information**

**
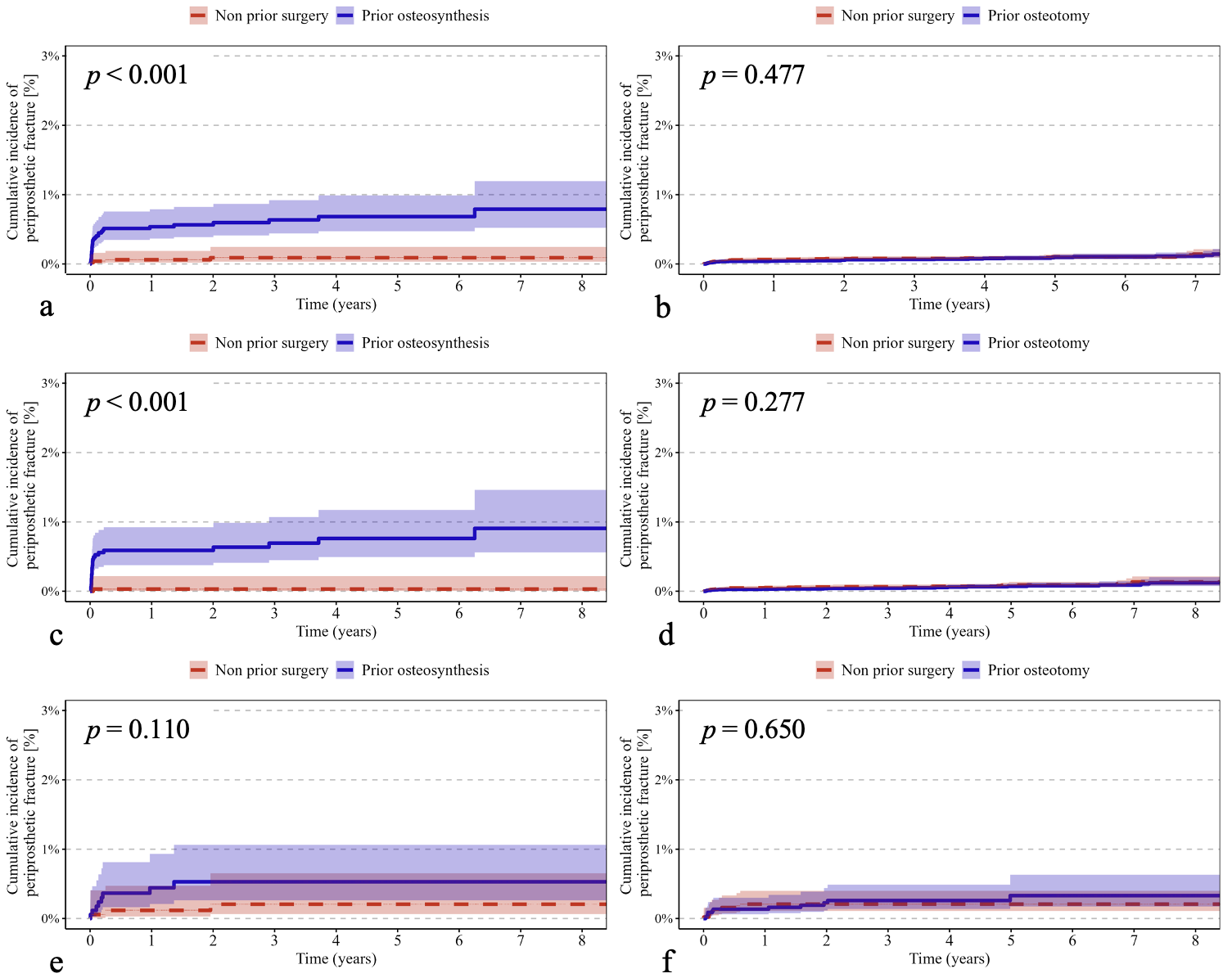
**

**Supplementary Fig. S1 Kaplan–Meier curves of cumulative incidence of periprosthetic fracture after TKA**
Comparison of patients with**prior surgery**(blue) vs**matched controls without prior surgery**(red) over**up to 8 years**. Panels:**(a)**all TKA after**osteosynthesis**(p < 0.001);**(b)**all TKA after **osteotomy**(p = 0.477);**(c) unconstrained**TKA after osteosynthesis (p < 0.001);**(d)**unconstrained TKA after osteotomy (p = 0.277); **(e) constrained**TKA after osteosynthesis (p = 0.110);**(f)**constrained TKA after osteotomy (p = 0.650). Shaded bands denote**95% confidence intervals (CI)**. p-values from the**log‑rank test.**

**
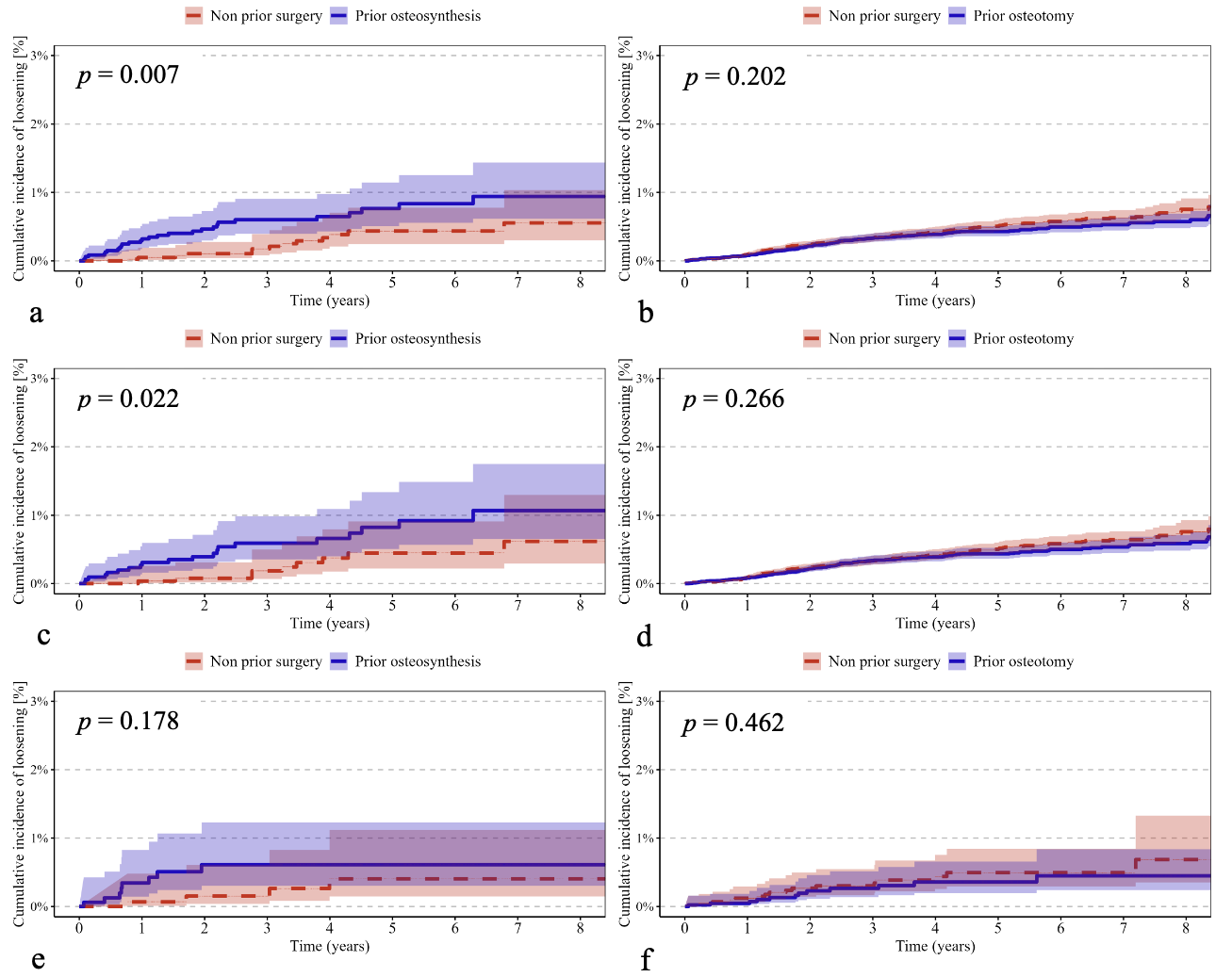
**

**Supplementary Fig. S2 Kaplan–Meier curves of cumulative aseptic loosening after TKA**
Comparison of patients with **prior surgery** (blue) vs **matched controls without prior surgery** (red) over **up to 8 years**. Panels: **(a)** all TKA after **osteosynthesis** (p = 0.007); **(b)**all TKA after **osteotomy**(p = 0.202); **(c) unconstrained**TKA after osteosynthesis (p = 0.022); **(d)**unconstrained TKA after osteotomy (p = 0.266); **(e) constrained**TKA after osteosynthesis (p = 0.178); **(f)** constrained TKA after osteotomy (p = 0.462). Shaded bands denote **95% confidence intervals (CI)**. p-values from the **log‑rank test.**
